# Sensitive Next-Generation Sequencing Method Reveals Deep Genetic Diversity of HIV-1 in the Democratic Republic of the Congo

**DOI:** 10.1128/JVI.01841-16

**Published:** 2017-02-28

**Authors:** Mary A. Rodgers, Eduan Wilkinson, Ana Vallari, Carole McArthur, Larry Sthreshley, Catherine A. Brennan, Gavin Cloherty, Tulio de Oliveira

**Affiliations:** a Infectious Disease Research, Abbott Diagnostics, Abbott Park, Illinois, USA; b Wellcome Trust-Africa Centre for Population Health, University of KwaZulu-Natal, Durban, Republic of South Africa; c School of Dentistry, University of Missouri—Kansas City, Kansas City, Missouri, USA; d Presbyterian Church (USA), Kinshasa, Democratic Republic of the Congo; e College of Health Sciences, University of KwaZulu-Natal, Durban, Republic of South Africa; f Research Department of Infection, University College London, London, United Kingdom; Ulm University Medical Center

**Keywords:** full-length genome, HIV-1 surveillance, next-generation sequencing, phylogenetic analysis, recombination, genetic diversity

## Abstract

As the epidemiological epicenter of the human immunodeficiency virus (HIV) pandemic, the Democratic Republic of the Congo (DRC) is a reservoir of circulating HIV strains exhibiting high levels of diversity and recombination. In this study, we characterized HIV specimens collected in two rural areas of the DRC between 2001 and 2003 to identify rare strains of HIV. The *env* gp41 region was sequenced and characterized for 172 HIV-positive specimens. The *env* sequences were predominantly subtype A (43.02%), but 7 other subtypes (33.14%), 20 circulating recombinant forms (CRFs; 11.63%), and 20 unclassified (11.63%) sequences were also found. Of the rare and unclassified subtypes, 18 specimens were selected for next-generation sequencing (NGS) by a modified HIV-switching mechanism at the 5′ end of the RNA template (SMART) method to obtain full-genome sequences. NGS produced 14 new complete genomes, which included pure subtype C (*n* = 2), D (*n* = 1), F1 (*n* = 1), H (*n* = 3), and J (*n* = 1) genomes. The two subtype C genomes and one of the subtype H genomes branched basal to their respective subtype branches but had no evidence of recombination. The remaining 6 genomes were complex recombinants of 2 or more subtypes, including subtypes A1, F, G, H, J, and K and unclassified fragments, including one subtype CRF25 isolate, which branched basal to all CRF25 references. Notably, all recombinant subtype H fragments branched basal to the H clade. Spatial-geographical analysis indicated that the diverse sequences identified here did not expand globally. The full-genome and subgenomic sequences identified in our study population significantly increase the documented diversity of the strains involved in the continually evolving HIV-1 pandemic.

**IMPORTANCE** Very little is known about the ancestral HIV-1 strains that founded the global pandemic, and very few complete genome sequences are available from patients in the Congo Basin, where HIV-1 expanded early in the global pandemic. By sequencing a subgenomic fragment of the HIV-1 envelope from study participants in the DRC, we identified rare variants for complete genome sequencing. The basal branching of some of the complete genome sequences that we recovered suggests that these strains are more closely related to ancestral HIV-1 strains than to previously reported strains and is evidence that the local diversification of HIV in the DRC continues to outpace the diversity of global strains decades after the emergence of the pandemic.

## INTRODUCTION

Multiple independent interspecies simian immunodeficiency virus (SIV) transmission events have resulted in the emergence of four major lineages of human immunodeficiency virus type 1 (HIV-1) in humans: groups N, O, and P and pandemic group M (1). Estimates place the emergence of the group M lineage of HIV-1 in the Congo Basin at the beginning of the 20th century (2, 3). For the purpose of this study, the Congo Basin includes the following countries: Angola, Cameroon, the Central African Republic, the Democratic Republic of the Congo (DRC), the Republic of the Congo, and Gabon. By 1959 or 1960, considerable HIV-1 diversity was already present in Kinshasa, DRC (4), where HIV-1 group M first emerged and then spread globally (5). The current nomenclature recognizes 9 major subtypes of HIV-1 group M (subtypes A to D, F to H, J, and K) and is entirely based on a phylogenetically based classification system. The most prevalent subtypes are A, B, C, D, and G, while subtypes F, H, J, and K are collectively responsible for only 1% of all infections worldwide (6). Subtypes H, J, and K are primarily found in West, South, and Central Africa, and only 2 to 7 complete genomes have been reported, making them extremely rare (6–8; Los Alamos National Laboratory [LANL] HIV Database). A subtype L was suggested to be a new classification on the basis of two distinct HIV-1 genomes collected in the DRC in 1983 and 1990; however, a third epidemiologically unlinked case has not been reported (9, 10). The sequences of many HIV-1 isolates from the Congo Basin, including those of the two putative subtype L genomes, do not cluster phylogenetically with other known sequences and are considered unclassified (9–13).

Another important genetic feature of HIV is that it is prone to recombination. High levels of intrasubtype diversity and intersubtype recombination are found in HIV-1 specimens from patients in the DRC (5, 11), a finding which is indicative of an old epidemic. Currently, 72 circulating recombinant forms (CRFs) have been identified in each of at least three unlinked HIV-1-infected individuals, while many more unique recombinant forms (URFs) have been described in one or two individuals (LANL HIV database). Recent analysis of whole-genome HIV-1 sequences from the Congo Basin identified fragments that clustered basal to all major subtypes, suggesting that the parental lineages of these recombinant fragments have not yet been sampled and characterized or that these strains have gone extinct and are no longer in circulation (12).

The diversity present in HIV specimens from the DRC and other countries within the Congo Basin makes these specimens a unique source for identifying rare and emerging variants; however, classification of these viruses is likewise complicated by the extreme genetic diversity observed in HIV variants within the region. Subtype-specific differences in treatment effectiveness, the development of resistance, vaccine coverage, and disease progression make surveillance and accurate classification of HIV-1 strains imperative (14–16). To date, most HIV-1 specimens from the DRC have primarily been classified by phylogenetic analysis of short partial genome sequences (400 to 900 bp) in either the group-specific antigen (*gag*), polymerase (*pol*), or envelope (*env*) gene of HIV-1 (4, 11, 13, 17). However, the full extent of recombination and sequence diversity of a complex genome cannot be completely characterized when partial genome sequences are used for HIV-1 classification. As a region with an exceptionally high level of HIV-1 strain diversity, this is especially true for specimens from the Congo Basin. Advances in next-generation sequencing (NGS) technologies, their availability, and the reduction in the cost of NGS technologies have enabled complete genome sequences to be used for the classification of HIV-1 specimens globally. Despite these advances, only 33 complete HIV-1 genomes from the DRC are currently available in the LANL HIV database. In contrast, 1,217 complete HIV-1 genomes from the United States are available in the LANL database, and 1,185 of these are classified as subtype B. Therefore, in order to fully characterize the true diversity of circulating HIV-1 strains, additional complete genomes of complex variants from the DRC must be sequenced.

As a part of ongoing surveillance efforts in sub-Saharan Africa, we have previously deposited 55 complete HIV-1 genomes from cultured virus isolates and Cameroonian patient specimens into the GenBank database (19–27). Recently, we developed a new HIV primer-specific NGS platform, called HIV-switching mechanism at the 5′ end of the RNA template (SMART; Clontech) (19) to obtain complete genomes from HIV-1 groups M, N, O, and P as well as HIV-2 isolates. This method utilizes a set of 6 pan-HIV-specific primers fused to the SMART sequence to create libraries for NGS on the Illumina MiSeq instrument (19). While this method is excellent for recovering genomes from diverse HIV sequences, it has limited success for clinical specimens with low viral loads (<5 log copies/ml). HIV-SMART was previously optimized for clinical specimens by adding a Benzonase pretreatment of the sample to digest background human DNA and RNA, and a direct correlation between the sample viral load and the resulting genome sequence coverage was observed (19). Increasing the total number or concentration of HIV-SMART primers did not have any benefit to genome coverage; however, an increase in the reverse transcription (RT) temperature from 42°C to 47°C improved the coverage depth, while RT at 52°C dramatically reduced the level of genome coverage. Alternative library preparation methods or the use of an RT temperature of between 47°C and 52°C may improve the levels of genome coverage and coverage depth for clinical samples with low viral loads, although these conditions have not been tested yet.

In the present study, we applied both Sanger-based *env* amplicon sequencing and HIV-SMART NGS to a set of HIV-1 variants from the DRC, resulting in 172 new *env* sequences and 14 new complete genomes. Modifications to the HIV-SMART method for samples with low viral loads by adding an input nucleic acid concentration step and lowering the RT temperature to 42°C improved the level of genome coverage to >90% for clinical samples with viral loads of >4 log copies/ml. Characterization of the complex *env* and complete genome sequences revealed a high level of HIV-1 diversity and recombination among HIV-1 isolates in the DRC and identified sequences that are outliers to known subtype and CRF sequences. Further analyses of the outlier sequences suggest that they may very well be ancestral to some of the major pandemic subtypes seen today.

## RESULTS

**FIG 1:**
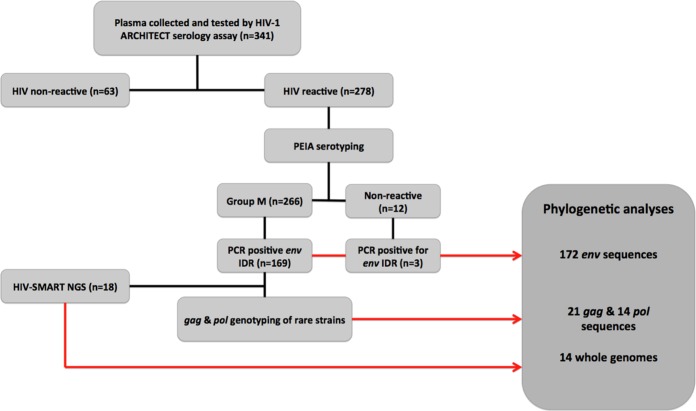
Specimen testing and genotyping work flow. The specimen processing and testing steps used in this study and the results are summarized in each box in the flowchart. The number (*n*) of specimens included in each step is indicated. Red arrows, genotypes used for phylogenetic analyses (described in the box with dark gray shading).

A total of 341 specimens were collected between 2001 and 2003 from study participants at the two rural study sites. Plasma screening by the Architect HIV Ag/Ab Combo (Abbott Diagnostics, Abbott Park, IL) serological assay identified 278 HIV-positive specimens (81.53%). Further serotyping with a peptide immunoassay (PEIA) classified the isolates in 266 of the HIV-positive specimens as belonging to group M of HIV-1 (95.68%), while 12 specimens (4.32%) were nonreactive for HIV-1 (Fig. 1).

**FIG 2:**
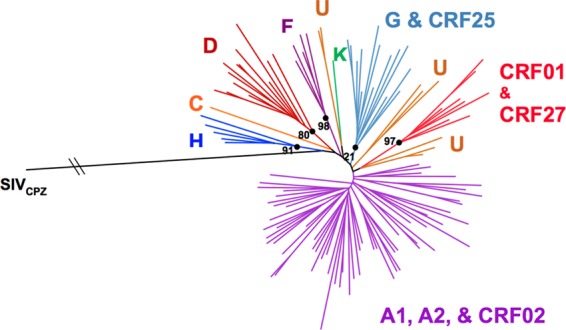
Neighbor-joining tree of 117 *env* IDR sequences. The tree was inferred by use of the Phylip software package. The tree was limited to a subset of sequences from 117 specimens representing the major identified classifications for better visualization. Bootstrap values are shown for major branch points, indicated by black dots.

RT-PCR and Sanger sequencing of the *env* immunodominant region (IDR) produced 172 *env* genotypes. Phylogenetic subtyping of *env* IDR genotypes identified the presence of eight of the nine major subtypes of HIV-1 group M, including rare subtypes H (*n* = 5), J (*n* = 3), and K (*n* = 1). The majority of isolates were classified as subsubtype A1 (*n* = 74, 43.023%), while four subsubtype A2 isolates were also identified. Subtypes D (*n* = 16, 9.302%) and G (*n* = 15, 8.721%) were the second and third most prevalent subtypes identified in the *env* IDR data set, respectively (Table 1). It is important to note that the use of a subgenomic region for subtyping, such as the *env* gp41, may miss several recombinants, and it is likely to overestimate the number of pure subtypes identified. In particular, subtype B isolates were not identified in our study population, although four CRFs (CRF01, CRF02, CRF25, and CRF27) were present (*n* = 20). A total of 20 sequences (11.63%) did not branch with reference sequences and were unclassified. A representation of the tree topology containing the 172 *env* IDR sequences from the DRC along with reference sequences is presented in Fig. 2.

**TABLE 1:** Subtype assignment of 172 *env* IDR sequences^*a*^

Subtype or CRF	No. of sequences	% of sequences
Subtype A1	74	43.023
Subtype A2	4	2.326
Subtype B	0	0
Subtype C	5	2.907
Subtype D	16	9.302
Subtype F1	8	4.651
Subtype F2	0	0
Subtype G	15	8.721
Subtype H	5	2.907
Subtype J	3	1.744
Subtype K	1	0.581
Subtype L	1	0.581
Unclassified	20	11.628
CRF01	6	3.488
CRF02	10	5.814
CRF25	2	1.163
CRF27	2	1.163
Total	172	100

a The total number of sequences and the percentage of the total of all sequences for each subtype are listed for the *env* region sequences. Unclassified sequences did not branch with references with bootstrap support of >70.

Sanger sequencing of the *gag* and *pol* regions of selected specimens produced 20 *gag* sequences and 14 *pol* sequences. Genotypes from *gag*, *pol*, and *env* were available for 11 specimens, while at least two genotypes from one of the three subgenomic regions were available for an additional 8 specimens. The genotyping results for the *gag* and *pol* subgenomic regions were cross-referenced with the *env* IDR genotypes and revealed eight possible recombinants. One isolate clustered basal to the two putative subtype L isolates in all three regions.

Eighteen isolates were selected for whole-genome sequencing on the basis of their subtype assignments in the *gag*, *pol*, and *env* data sets. The HIV viral load for the selected specimens ranged from 3.89 to 5.82 log_10_ copies/ml (Table 2). The HIV-SMART NGS method (19) developed by our group has previously been shown to generate complete genomes from clinical samples with viral loads of greater than 5 log_10_ copies/ml; however, this method has not been applied to clinical specimens with viral loads below this cutoff. Therefore, the specimens from DRC with viral loads of <5 log_10_ copies/ml were used in HIV-SMART NGS optimization experiments as samples with low viral loads.

**TABLE 2:** Summary of the 18 isolates that were chosen for whole-genome sequencing using the HIV-SMART method^*a*^

Isolate	Viral load (log_10_ no. of copies/ml)	*gag* subtype	*pol* subtype	*env* IDR subtype	Genome coverage (%)	Genome length (no. of nucleotides)
NGSID 1	5.26			C	100	9,692
NGSID 2	4.35	C	C	C	100	9,723
NGSID 3	4.45			D	100	9,751
NGSID 4	4.02			F1	75	9,459
NGSID 5	4.65			F1	100	9,743
NGSID 6	5.38			U	100	9,660
NGSID 7	5.28			CRF25	100	9,764
NGSID 8	5.2	PCR neg	PCR neg	U	100	9,556
NGSID 9	4.29	A1	A1	H	72	9,633
NGSID 10	5.01	U	PCR neg	U	100	9,621
NGSID 11	3.89	L	L	L	63	8,872
NGSID 12	5.2	K	K	K	99.75	9,579
NGSID 13	5.42	J	J	J	100	9,688
NGSID 14	5.82	H	H	H	100	9,716
NGSID 15	5.24	H	H	H	100	9,734
NGSID 16	4.7	H	H	H	100	9,658
NGSID 17	4.36	A1		H	67	8,649
NGSID 18	4.75	A1	PCR neg	J	100	9,622

a The viral load was quantified by a RealTime HIV-1 assay (Abbott Molecular Diagnostics). Subtyping of *gag*, *pol*, and *env* IDR sequences was performed through maximum likelihood phylogenetic inference of a 468-bp region of *gag*, an 864-bp region of *pol*, and a 676-bp region of *env*, respectively. The whole-genome coverage and genome length were calculated with CLC Bio software for the final consensus genome sequences that were generated using the HIV-SMART sequencing method. neg, negative.

**FIG 3:**
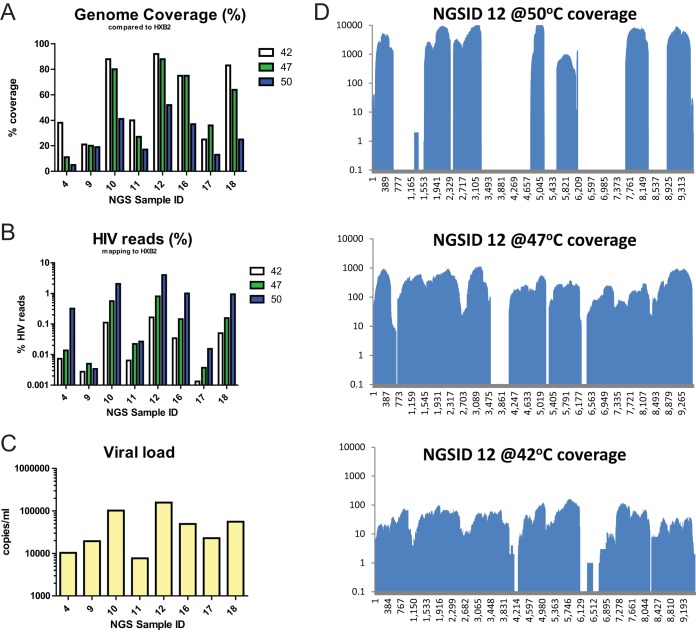
Reverse transcription temperature optimization. The trimmed NGS reads from HIV-SMART libraries prepared at the indicated reverse transcription temperature (42°C, 47°C, or 50°C) were mapped to the HXB2 reference genome. (A, B) The genome coverage (A) and percentage of HIV reads (B) for this alignment were calculated by use of the CLC Bio software. (C) The viral load for each sample tested is plotted on a log scale. (D) The genome coverage plots for each position of the genome are shown for NGSID 12, which showed a trend representative of the trends seen in all other samples tested. *y* axis, number of reads; *x* axis, nucleotide position in the genome sequence.

**FIG 4:**
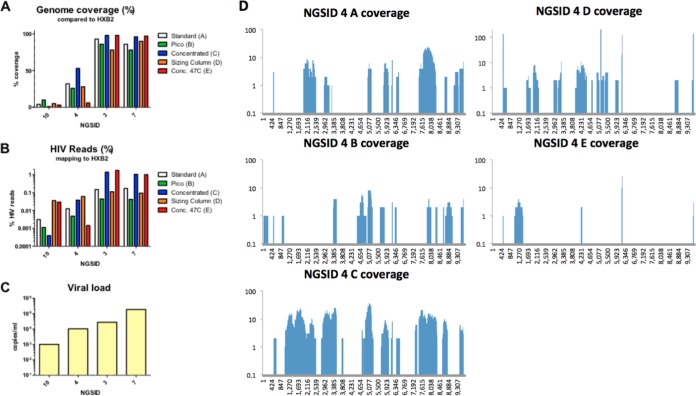
Library preparation optimization. The trimmed NGS reads from HIV-SMART libraries were prepared by protocols A to E (A, the standard protocol; B, a protocol with the Pico SMART cDNA kit; C, a protocol with a nucleic acid concentrator; D, a protocol with a sizing column; E, a protocol that followed the nucleic concentration protocol [Conc.] in protocol C with reverse transcription at 47°C) and mapped to the HXB2 reference genome. (A, B) The genome coverage (A) and percentage of HIV reads (B) were calculated for this alignment by the use of CLC Bio software. (C) The viral load for each sample tested is plotted on a log scale. (D) The genome coverage plots for each position of the genome are shown for isolate NGSID 4, which showed a trend representative of the trends seen for all other isolates tested. *y* axis, number of reads; *x* axis, nucleotide position in the genome sequence.

Several conditions were tested to improve the genome coverage and read depth of clinical specimens with low viral loads. First, the RT temperature was raised to 47°C or 50°C to improve the primer binding specificity. Second, a larger amount of input RNA was used to make the cDNA libraries by concentration of nucleic acid extracts either on a concentrator filter column (Zymo Research) or by using a Pico SMART cDNA kit (Clontech). Third, a sizing column (Clontech) was used to select larger amplicons from the total SMART cDNA libraries in a cleanup step. Lastly, the 47°C RT condition was combined with the use of the concentrator column. In the RT temperature comparison, 8 specimens with viral loads ranging from 3.89 to 5.2 log_10_ copies/ml were included, and 7 had the highest read depth at 50°C and the highest percentage of HIV reads at 42°C (Fig. 3). Notably, the genome coverage was approximately 50% lower in the libraries with which RT was conducted at 50°C than the libraries with which RT was conducted at 42°C (Fig. 3). Therefore, an RT temperature of 42°C was selected for specimens with low viral loads. In the library preparation optimization experiments, 4 samples covering a viral load range of 3 to 5 log_10_ copies/ml were included. All conditions that included the nucleic acid concentrator step had the highest genome coverage and the highest percentage of HIV reads for specimens with viral loads of >4 log_10_ copies/ml (Fig. 4). For these specimens, raising the RT temperature to 47°C did not affect genome coverage when the concentrator was used. Specimens with viral loads of <4 log_10_ copies/ml had inconsistent levels of genome coverage, percentages of HIV reads, and read depths for all of the tested conditions. These results indicate that addition of the concentrator step greatly improved the quality and coverage of genome sequences that can be generated by HIV-SMART.

The HIV-SMART NGS method with an RT temperature of 42°C and a nucleic acid concentrator step for specimens with low viral loads resulted in complete genome sequences (>99% coverage) for 14 specimens and partial genomes for 4 specimens (Table 2). All complete genomes had a length of at least 9,550 nucleotides, and the average coverage depth was >10 reads for all of the regions sequenced by HIV-SMART NGS (see Fig. S1 in the supplemental material). Several short gaps (<200 bp) were filled in by Sanger sequencing to complete 5 genomes. The 4 partial genomes had coverages ranging from 63% to 75%, with gaps of various sizes occurring throughout the sequences (Table 2 and Fig. S1). The 14 complete genomes produced by HIV-SMART NGS were subsequently classified by phylogenetic inference and recombinant analysis.

Online subtyping tools were initially used to classify the 14 whole genomes from the DRC (Table 3). The REGA (v3.0) subtyping tool identified seven isolates of pure viral subtypes, including one subtype C isolate (NGSID 2), one subtype D isolate (NGSID 3), one subtype F1 isolate (NGSID 5), one subtype J isolate (NGSID 13), and three subtype H isolates (NGSID 14 to NGSID 16). The remaining seven whole-genome sequences from the DRC were classified as possible recombinants. Subtyping by use of the jumping profile hidden Markov model (jpHMM) confirmed the classification of pure viral isolates identified with REGA (v3.0). Additionally, jpHMM classified NGSID 1 as a pure subtype C isolate, whereas REGA (v3.0) classified this isolate as a recombinant form between subtypes C and D. REGA (v3.0) and jpHMM identified broadly similar recombinant profiles for the six putative recombinant genomes (Table 3).

**TABLE 3:** Results of online subtyping methods^*a*^

Isolate	REGA (v3.0)		jpHMM	
	Classification	Bootstrap support (%)	Classification	Posterior probability
NGSID 1	Recombinant of subtypes C and D	NA	Subtype C	1
NGSID 2	HIV-1 subtype C	100	Subtype C	1
NGSID 3	HIV-1 subtype D	100	Subtype D	1
NGSID 5	HIV-1 subtype F1	100	Subtype F1	1
NGSID 6	Recombinant of subtypes G, A1, and H	NA^*b*^	Recombinant of subtypes A1, G, and H	0.8–1.0
NGSID 7	Recombinant of subtypes 25_cpx, A1, and G	NA	Recombinant of subtypes A1 and G	0.7–1.0
NGSID 8	Recombinant of subtypes H, A1, 04_cpx, G, and K	NA	Recombinant of subtypes A1, H, and K	0.9–1.0
NGSID 10	Recombinant of subtypes H, A1, 04_cpx, G, and K	NA	Recombinant of subtypes A1, H, and K	0.9–1.0
NGSID 12	Recombinant of subtypes K, J, and F1	NA	Recombinant of subtypes C, F1, and K	0.6–1.0
NGSID 13	HIV-1 subtype J	100	Subtype J	1
NGSID 14	HIV-1 subtype H	100	Subtype H	1
NGSID 15	HIV-1 subtype H	100	Subtype H	1
NGSID 16	HIV-1 subtype H	100	Subtype H	1
NGSID 18	Recombinant of subtypes A1, J, K, and G	NA	Recombinant of subtypes A1, J, and K	0.6–1.0

a Fourteen whole-genome sequences were subtyped by the REGA (v3.0) and jpHMM online subtyping methods.

b NA, not applicable.

**FIG 5:**
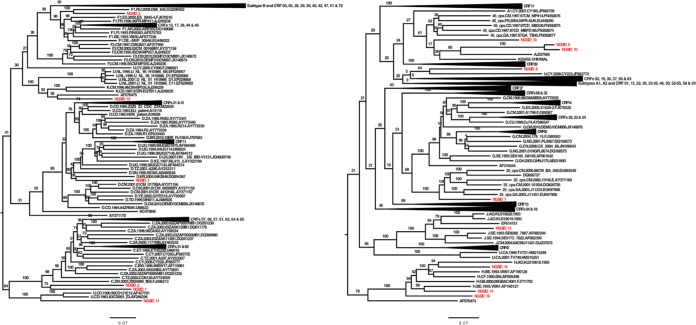
ML phylogenetic tree of 14 whole-genome sequences from the DRC (highlighted in red) and a representative number (*n* = 485) of HIV-1 reference strains. The tree was constructed with the RAxML (v8.0.0) program, the GTR+G model, and 1,000 bootstrap replicates. The bar at the bottom represents the genetic distance along branches. This tree was rooted at the midpoint, and branches for major clades that did not cluster with isolates from the DRC were collapsed. Bootstrap values for major splits in the tree topology are shown in individual clades.

Subtyping of the genomes through manual phylogenetic inference supported the classification for the eight isolates that were classified as pure subtypes by NGS (Fig. 5 and Table 4). Isolates NGSID 1 and NGSID 2 clustered basal to the main subtype C clade and also contained major C subtype-like CRFs (e.g., CRF07 and CRF08) with good bootstrap support. Given the clustering of recombinants within the main subtype C clade and the basal clustering of isolates NGSID 1 and NGSID 2 to the main subtype C clade, there is a good probability that small recombination events might have occurred in these two specimens and that these might not have been picked up by the online subtyping tools. NGSID 3 clustered within the main subtype D clade, while NGSID 5 clustered along with a subtype F1 isolate from Russia (GenBank accession number GQ290462) basal to the main subtype F1 clade. NGSID 6 clustered with one unclassified isolate (GenBank accession number JF683772), though the split in the tree topology separating these two isolates from the rest of the tree was not supported (Fig. 5 and Table 4). These two isolates in turn clustered basal to the main subtype G clade, which is indicative of possible recombination in these two isolates.

**TABLE 4:** Subtype assignment of the 14 genotypes from the DRC obtained by NGS^*a*^

Isolate	Simplot		Bootscan		Large phylogeny	
	Classification	Support (% similarity)^*b*^	Classification	Support (% of permutated trees)^*c*^	Classification	Bootstrap support (%)
NGSID 1	Majority subtype C	0.80–0.94	Majority subtype C	22–100	Outlier subtype C	100
NGSID 2	Majority subtype C	0.83–0.95	Majority subtype C	48–100	Outlier subtype C	100
NGSID 3	Majority subtype D	0.93–0.97	Majority subtype D	42–100	Subtype D	89
NGSID 5	Majority subtype F1	0.87–0.98	Majority subtype F1	74–100	Subtype F1	98
NGSID 6	Subtype A1/G/A1/G/H/G	NA^*d*^	Subtype A1/G/A1/G/H/G	NA	Unclassified URF	64
NGSID 7	Subtype A1/G/A1/G/A1/G	NA	Subtype A1/G/A1/G/A1/G	NA	Basal to 25_cpx	100
NGSID 8	Subtype A1/K/A1/H/A1	NA	Subtype A1/K/A1/H/A1	NA	Unclassified URF	80
NGSID 10	Subtype A1/K/A1/H/A1	NA	Subtype A1/K/A1/H/A1	NA	Unclassified URF	80
NGSID 12	Subtype K/F1/K/F1/K	NA	Subtype K/F1/K/F1/K/G	NA	Unclassified URF	92
NGSID 13	Majority subtype J	0.82–0.96	Majority subtype J	84–100	Outlier subtype J	100
NGSID 14	Majority subtype H	0.79–0.98	Majority subtype H	82–100	Subtype H	100
NGSID 15	Majority subtype H	0.86–0.99	Majority subtype H	74–100	Subtype H	100
NGSID 16	Majority subtype H	0.86–0.96	Majority subtype H	96–100	Outlier subtype H	100
NGSID 18	Subtype A1/K/A1/J/A1	NA	Subtype A1/K/A1/J/A1	NA	Unclassified URF	17

a The subtype classifications for the 14 genotypes obtained by NGS made by the use of Simplot software, bootscan analysis, and manual phylogenetic inference are presented.

b Lowest to highest range of similarity.

c Lowest to highest range of the percentage of permutated trees.

d NA, not applicable.

Isolate NGSID 7 clustered basal to a cluster containing isolates belonging to CRF25_cpx with good support. Contained within this CRF25_cpx cluster was one problematic isolate (GenBank accession number DQ826727). Closer investigation revealed this isolate to be a complex unique recombinant form between CRF02 and CRF25. NGSID 8 and NGSID 10 clustered basal to the clade containing CRF04, though the branch separating these two isolates from the CRF04 clade was very long, which is indicative of a substantial genetic distance between the DRC isolates and CRF04. NGSID 12 clustered with another unclassified isolate from the reference data set (GenBank accession number AF076475) with 92% bootstrap support for the split separating these two isolates from the rest of the tree topology. The unclassified isolate from the reference data set (GenBank accession number AF076475) is a unique recombinant form between subtypes F2 and K and contains unclassified regions, and it was characterized from an individual from Belgium. The basal clustering of NGSID 12 along with the unclassified isolate from the reference data set (GenBank accession number AF076475) with the main subtype K clade is indicative of possible recombination in these two isolates. NGSID 13 clustered within the subtype J branch along with the newly characterized subtype J isolates from Angola (8). NGSIDs 14 and 15 clustered within the main subtype H clade, while NGSID 16 clustered basal to the main subtype H clade. The split separating these three isolates and the subtype H clade from the rest of the tree topology was very well supported. Finally, NGSID 18 clustered basal to a clade containing CRF45_cpx, though the branch of NGSID 18 was long, which indicates a substantial genetic distance between NGSID 18 and CRF45 isolates.

Recombination analyses were performed to investigate putative recombinants within our data set. Recombination Detection Program, v4.0 (RDP4), analyses of the eight pure subtype isolates identified small possible recombination events, although none of the *P* values for these classifications were significant. Isolate NGSID 6 was classified as a recombinant form between subtypes A1, G, and H. Notably, RDP4 indicated that the subtype H fragment of NGSID 6 was more closely related to the homologous subtype H segments of NGSIDs 8 and 10 than to other subtype H reference sequences. In the RDP4 phylogenetic tree inference, NGSIDs 6, 8, and 10 clustered basal to the main subtype H clade, while NGSIDs 14 to 16, which were the three pure subtype H whole-genome isolates, clustered within the main subtype H clade. RDP4 classified the majority of the viral backbone of NGSID 7 as belonging to CRF25_cpx, with a small recombinant fragment in the *env* region corresponding to subtype A1.

Similar recombination profiles for isolates NGSID 8 and NGSID 10 were uncovered by RDP4. RDP4 classified these two isolates as recombinants between subtypes A1, G, and H. RDP4 classified NGSID 12 as a pure subtype K isolate with no sign of recombination. Finally, RDP4 classified NGSID 18 as a recombinant between subtypes A1, K, and J.

**FIG 6:**
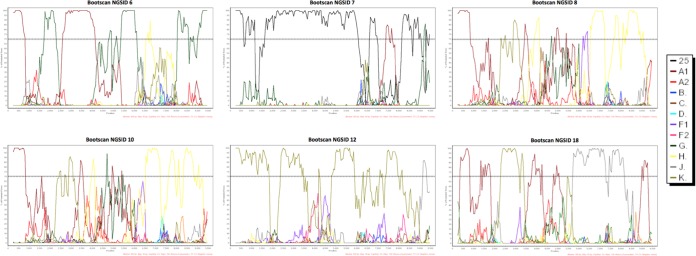
Bootscan plots of the six recombinant whole-genome sequences from the DRC. Each bootscan plot was performed with the Kimura-2 model of nucleotide substitution with a window size of 500 and a step size of 50. The color-coded key represents the different subtypes, subsubtypes, and CRFs of HIV-1. The dotted lines represent 70% of permutated trees.

Manual bootscan analyses that were performed on the 14 whole-genome sequences from the DRC supported the classification of the eight pure viral subtypes. The recombinant breakpoints that were uncovered by bootscan analyses of the six putative recombinant isolates (Fig. 6) broadly reflected trends similar to those obtained by the RDP4 analyses as well as those from the online subtype methods.

**FIG 7:**
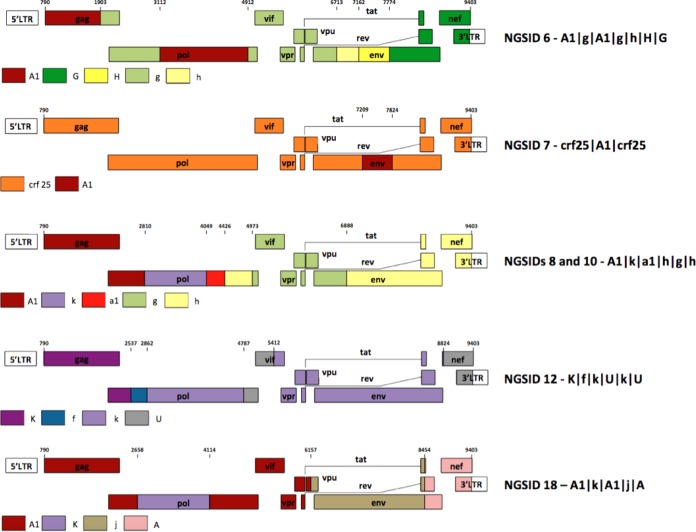
Recombinant mosaic profiles of the six recombinant whole-genome sequences that were generated in the course of this study. The numbers at the top of each genome represents the recombinant breakpoints relative to the HXB2 reference strain. The recombinant profile of each isolate is presented on the right-hand side, with capital letters referring to clustering within established clades and lowercase letters representing basal clustering to that particular clade.

Phylogenetic inference of recombinant fragments was used to classify each of the six recombinant genomes in our sequence cohort (Fig. S2 to S6). Isolate NGSID 6 was classified as a complex recombinant form between subtypes A1, G, and H with the following mosaic structure: A1|g|A1|g|h|H|G, where lowercase letters are used to classify recombinant fragments derived from a virus that branches basal to a given subtype in a phylogenetic tree and capital letters indicate that the fragment clusters within the currently known subtype diversity (Fig. S2). Analyses of NGSID 7 classified this isolate as a recombinant between CRF25 and subsubtype A1. Notably, the segment of the genome corresponding to CRF25 clustered basal to the main CRF25 clade, which resulted in the following mosaic recombinant structure for this isolate: crf25|A1|crf25 (Fig. S3). The six recombinant viral genomes and their respective mosaic structures are presented in Fig. 7.

Our manual phylogenetic inference of recombinant fragments in isolates NGSID 8 and NGSID 10 suggests that these two isolates are identical recombinants. The tree inference of recombinant fragments indicates that these two isolates are complex recombinants between subtypes A1, G, H, and K with the following mosaic structure: A1|k|a1|h|g|h (Fig. S4). Our tree inference of NGSID 12 classified this isolate as a recombinant form between subtypes K and F. However, the subtype F fragment could not be unambiguously classified as belonging to either subsubtype F1 or F2, as this isolate clustered basal to the main subtype F clade. An additional two segments, one in the 3′ *pol* and *vif* region and the other in the *nef* and 3′ long terminal repeat (LTR) region, could not be classified due to poor branch support and were subsequently categorized as unclassified. The mosaic structure of NGSID 12 was assigned as follows: K|f|k|U|k|U (Fig. S5). Finally, NGSID 18 was classified as a complex recombinant between subtypes A1, J, and K. The recombinant fragment on the 3′ LTR side of this isolate clustered with both subtype A1 and A2 isolates, and the isolate was subsequently categorized as subtype A. The final mosaic structure for this isolate was as follows: A1|k|A1|j|A (Fig. S6).

## DISCUSSION

The complete and subgenomic HIV-1 sequences characterized in the present study have considerably expanded the known genetic pool of HIV-1 strains from the DRC and improved our understanding of the epidemic within the Congo Basin. The characterization of 172 *env* IDR genotypes identified a wide pool of genetic variants, with subtypes A1, D, and G being the most frequently identified within the cohort. Small numbers of rare viral subtypes were also identified, including subtypes A2 (*n* = 4), H (*n* = 5), J (*n* = 3), and K (*n* = 1). A small number of CRFs were also identified, including CRF01_AE (*n* = 6), CRF02_AG (*n* = 10), CRF25_cpx (*n* = 2), and CRF27_cpx (*n* = 2) (Table 1 and Fig. 2). Initial subtyping was inferred by analyzing *env* gp41 sequences rather than whole-genome sequences. This approach may fail to detect recombinants and overestimate the number of pure subtypes. Of the 172 specimens whose *env* IDRs were sequenced, either the *gag* or *pol* region of 19 specimens was also sequenced. All three regions of one isolate clustered basal to two unclassified isolates which were previously suggested to be a new subtype of HIV-1 group M (subtype L). Cross-referencing of the *gag* and *pol* genotypes with the *env* IDR genotypes identified eight possible recombinants. However, the true number of recombinants in our study cohort is most likely much higher, given the small number of patients for whom more than one region of the genome of their HIV-1 isolates was sequenced.

Appreciation of the genetic diversity observed in subgenomic sequences prompted our efforts to sequence the complete genomes of the rare variants identified in the *gag*, *pol*, and *env* regions. Unfortunately, many of the rare variants that we selected had low viral loads upon quantitation and failed to produce RT-PCR bands for other regions of the HIV-1 genome for sequencing. Therefore, to efficiently sequence the 18 selected samples, we followed an NGS method that was optimized for samples with low viral loads. Improvement of the HIV-SMART NGS method resulted in complete or nearly complete genome sequences for samples with viral loads below the previous threshold of 5 log_10_ copies/ml. Despite the comparison of six different conditions to improve genome coverage and read depth, clinical specimens with viral loads that were less than 4 log_10_ copies/ml were not consistently sequenced by the modified HIV-SMART method. The reduction in reliable complete genome coverage for these specimens with low viral loads is likely due to poor HIV-SMART primer annealing, extension, or a combination of the two. For samples with viral loads below this threshold, sequencing of libraries of purified RT-PCR amplicons may be an alternative method that could reduce the number of background reads and improve the read depth. Of the conditions tested, the lowest RT temperature (42°C) and the addition of a nucleic acid concentration step before library preparation produced the greatest improvements in genome coverage and read depth (Fig. 3 and 4). While higher temperatures dramatically improved the read depth in this study, overall genome coverage was reduced by nearly half, suggesting that the reverse transcriptase enzyme was less processive or that the RNA template was degraded at higher temperatures, despite expectations that genome coverage would improve due to increased denaturation of RNA template secondary structures (28) (Fig. 4). Although the Pico SMART cDNA synthesis kit accommodated a larger volume of input nucleic acid template than other library preparation methods, it did not improve the genome coverage or read depth obtained by NGS (Fig. 4). Removal of short transcripts by the use of a sizing column was expected to improve the signal-to-background ratio for HIV-1 reads, but this step did not affect the percentage of HIV reads (Fig. 4). In contrast, the concentration of the input nucleic acid greatly improved the sequencing results (Fig. 4), which may have been due to both the concentration and the purification functions of the columns. The addition of the concentrator column may also improve the read depth for samples with high viral loads, although this has not been tested. The complexity of the genomes obtained from patient samples by the modified HIV-SMART NGS protocol is an excellent example of the utility of this method for surveillance of diverse HIV-1 sequences. The application of HIV-SMART NGS to larger quantities of samples will ultimately bring viral surveillance to the whole-genome scale and improve our understanding of the true diversity and evolution of HIV at the inter- and intrapatient levels. Ultimately, the optimized HIV-SMART NGS method combined with Sanger sequencing of short gap regions resulted in complete genome sequences for 14 rare variant HIV-1 specimens and 4 partial genomes (Table 2). We plan to use this important data set in the future to identify the date of origin of subtypes C, H, and J. Future incorporation of Primer ID sequences into the HIV-SMART method will allow the analysis of the intrapatient diversity of rare HIV specimens from the Congo Basin.

The characterization and classification of the 14 isolates from the DRC whose whole genomes could be sequenced were complicated by the extreme genetic diversity observed within our sample cohort and sequence data set. In particular, several of the viral isolates of pure subtypes and recombinant fragments clustered basal to major HIV-1 group M clades. In the majority of cases, this basal clustering was due to the small number of viral isolate genomes with which the new genotypes could be compared. For example, any analysis by comparison with the genome of subtype K isolates is limited to only two whole-genome reference sequences. This resulted in the basal clustering of any of the subtype K recombinant fragments identified in NGSIDs 8, 10, 12, and 18. A similar basal clustering of recombinant fragments corresponding to subtypes A1, G, J, and H and CRF25 was observed in the recombinants described here. Additionally, one of the subtype H isolates and the two subtype C isolates described here also clustered phylogenetically basal to their respective subtype clades. This basal clustering of sequences from the DRC underscores the deep genetic diversity of the isolates responsible for the global pandemic that are still circulating in relatively high numbers in areas within the Congo Basin.

**FIG 8:**
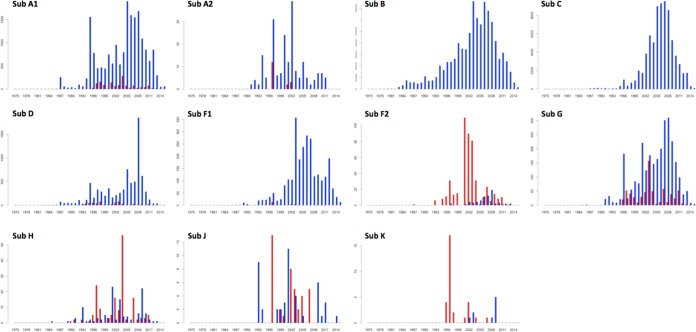
Frequency of sampling of major subtypes of HIV-1. Red bars, sampling within the Congo Basin; blue bars, sampling outside the Congo Basin. The *y* axes are not proportional. The numbers on the *y* axes represent the number of genotypes (>500 bp), while the numbers on the *x* axes represent years in calendar time. Sub, subtype.

Although it is clear that HIV-1 originated in the Congo Basin (2, 29), we still know little about the early transmission and dissemination of the strains that left the Congo Basin to cause epidemics outside that region. In order to test the hypothesis that much of the genetic diversity did not leave the Congo Basin, we analyzed all of the public sequences in the LANL database (date of access, 8 April 2016). This data set included all of the HIV-1 group M subtype sequences and CRF sequences which were >500 bp. In total, we found 411,194 sequences, 7,158 (1.74%) of which were from the Congo Basin. The Congo Basin still contains most of the diversity of HIV-1 in the world (6, 30). Subtypes that caused large epidemics outside the Congo Basin are still prevalent in this region, including subtypes A1, D, and G (Fig. 8).There are also subtypes in the Congo Basin that have not caused significant epidemics outside the region, such as subtypes A2, F2, H, J, and K. In total, 1,160 sequences of these subtypes have been identified to date, and 721 (62.15%) of these were identified in the Congo Basin. Furthermore, most of the subtype H, J, and K strains that have been identified outside the Congo Basin are from expatriates or from visitors to the region (28, 31–34).

Interestingly, the two main epidemiologically important subtypes in the world originated in the Congo Basin but are now not commonly found in the region (6, 30). The LANL public data set contained only 18 subtype B sequences and 14 subtype C sequences from this region. We also did not identify any subtype B isolates in our samples. This supports the hypothesis that the subtype B ancestral strain left the Congo Basin at an early stage of the HIV pandemic (4). However, we were able to identify subtype C in five samples and managed to sequence the first two whole genomes of subtype C isolates from the Congo Basin. In our phylogenetic reconstruction, the whole genomes of these two subtype C isolates clustered basal to all known subtype C sequences (Fig. 5). Their basal clustering suggests that these sequences are ancestors of the global subtype C pandemic. Our results are supported by those of other epidemiological and phylogenetic studies that used subgenomic regions of subtype C (5, 35).

The current nomenclature system for HIV-1 needs to be updated in order to track epidemiologically important strains. The current nomenclature recognizes nine subtypes (subtypes A, B, C, D, F, G, H, J, and K), four subsubtypes (subsubtypes A1, A2, F1, and F2), and almost 100 CRFs (74 CRFs at the time of writing of this paper). The majority of the subtypes, subsubtypes, and CRFs have very limited epidemiological importance (6). For example, as shown in this report, subtypes A2, F2, H, J, and K are mostly restricted to the Congo Basin (Fig. 8). We have also estimated that only 10 of the 74 CRFs in the LANL public data set seem to be epidemiologically important by plotting their distribution over time (i.e., more than 50 sequences sampled over 5 years). These include CRF01_AE, which is currently spreading in Southeast Asia; CRF02_AG, which is currently spreading in West Africa; CRF07_BC, which is currently spreading in China; CRF18_cpx and CRF19_cpx, which are currently spreading in Cuba; CRF35_AD, which is currently spreading in Afghanistan; and CRF63_02A1, which is currently spreading in Russia.

We suggest that in the future the HIV-1 nomenclature system annotate only strains of epidemiological importance. This would be especially valuable for new CRFs, as otherwise, high-throughput NGS methods, such as the one described in this report, will end up identifying 100s of new CRFs, which will further complicate the current nomenclature system. Focusing on the most epidemiologically important strains may facilitate the development of more effective HIV drugs and vaccines. For example, focused research on subtype C, which accounts for over 50% of the global infections, is crucial. Recent results suggest that the K65R mutation, one of the main mutations causing resistance to the first-line antiretroviral drug tenofovir, emerges rapidly in subtype C isolates (36, 37).

We also suggest that recombinant fragments of the genome be named according to the scheme introduced by Tongo and colleagues (12). This nomenclature system uses lowercase letters to classify recombinant fragments derived from a virus that branches basal to a given subtype in a phylogenetic tree and capital letters to represent fragments clustering within the currently known subtype diversity. For example, when we use this system to annotate our recombinants, NGSID8 was classified as A1|k|A1|j|A. This system may be particularly useful for the discovery and characterization of more of the highly divergent lineages that exist in the Congo Basin. This may shed light on specific viral genetic factors that enabled HIV-1 group M strains to leave the Congo Basin and cause major worldwide epidemics.

### Conclusions.

The advances in next-generation sequencing methods, such as the one presented in this study, can be used to sequence rare and diverse HIV-1 samples. Here, we identified new subtypes and recombinants that expand the genetic diversity of HIV-1 in the Congo Basin, which is the region where HIV-1 originated. The basal branching of some of the subtypes and recombinant segments that we recovered shows that these strains are more closely related to ancestral HIV-1 sequences than to the sequences of previously reported strains. It is evidence that the local diversification of HIV-1 in the Congo Basin continues to outpace the diversification of global strains. With an improved understanding of HIV-1 genetic diversity, we will be better able to assess the risks of the emergence of future outbreaks, to track the evolution of the global pandemic, and to develop new drugs and vaccine targets.

## MATERIALS AND METHODS

### Specimens.

Plasma specimens were collected at the Vanga Hospital, Bandundu Province, DRC, and The Good Shepherd Hospital, located 12 km from Kananga, Kasia-Occidental Province, in the DRC between 2001 and 2003. The specimens came from participants seeking voluntary testing and pregnant women participating in a prevention of mother-to-child transmission (pMTCT) program. Samples were acquired according to the 98-041e protocol, approved by the University of Missouri—Kansas City Research Board. We used a progressive analytical approach to test specimens and to identify rare viral subtypes circulating within our study population. A schematic breakdown of this analytical approach is illustrated in the flow diagram in Fig. 1.

### Serology.

Briefly, specimens were initially tested using the Architect HIV Ag/Ab Combo assay (Abbott Diagnostics, Abbott Park, IL) in order to identify HIV-infected specimens. The viral load of selected reactive specimens was quantified by an Abbott real-time HIV-1 assay (Abbott Molecular, Des Plaines, IL) according to the manufacturer's instructions. HIV-reactive specimens were serotyped with a research-use-only peptide immunoassay (PEIA) in order to classify specimens on the basis of HIV type (HIV-1 or HIV-2) and group (M, N, O, or P). Synthetic peptides specific for the *env* IDR of gp41 and *env* gp120 V3 from HIV-1 groups M, O, N, and P, HIV-2, and two strains of SIV_CPZ_ (chimpanzee) and SIV_RCM_ (red-capped mangabey) were covalently coupled separately to Luminex MagPlex beads before dilution into buffer (1% bovine serum albumin [BSA] in phosphate-buffered saline [PBS]). A round-bottom polystyrene white 96-well plate (Costar) containing 50 μl of a bead mixture and 50 μl of sample in each well was incubated for 30 min at room temperature in a plate shaker at 300 rpm. Liquid was aspirated using a BioTek 405 TS magnetic plate washer, and wells were washed with ∼250 μl of PBS–Tween 20 wash buffer (BioTek, Shoreline, WA). Fifty microliters of 0.4 μg/ml biotinylated goat anti-human IgG (Sigma, St. Louis, MO) was added to each well, and the plates were incubated for 15 min at room temperature in a plate shaker. After washing, 50 μl of 0.4 μg/ml streptavidin–R-phycoerythrin conjugate (SAPE; Invitrogen, Carlsbad, CA) was added to each well, and the plates were incubated for 10 min at room temperature in a plate shaker. After final washes, the beads were resuspended in 150 μl of reading buffer (1% BSA in PBS) and analyzed on a Luminex FlexMap 3D instrument (Luminex Corp. Austin, TX). For each bead set, ∼100 events were read, and results are expressed as the median fluorescence intensity (MFI) per 100 beads.

### RNA extraction.

Following serological classification, nucleic acid was extracted from samples according to the manufacturers' instructions using either (i) a QIAcube blood and body fluid spin protocol (Qiagen) or (ii) a research-use-only total nucleic acid sample preparation protocol on an m2000sp system (Abbott Molecular, Des Plaines, IL). For NGS experiments, plasma was pretreated with Benzonase before nucleic acid extraction. One-tenth volume of 10× Benzonase buffer (200 mM Tris-HCl, pH 7.5, 100 mM NaCl, 20 mM MgCl_2_) and 250 units/ml of ultrapure Benzonase (Sigma, St. Louis, MO) were added to 0.9 volume of plasma to degrade the free DNA and RNA (39). Samples were incubated at 37°C for 3 h and then filtered by centrifugation (5,000 rpm) through 0.22-μm-pore-size spin filters (Millipore, Billerica, MA) before extraction. For samples with low viral loads, the postextraction concentration of 25 to 50 μl of eluted nucleic acid was determined by using a concentrator column kit (Zymo Research) following the manufacturer's instructions.

### Subgenomic sequencing.

Reverse transcription (RT)-PCR and Sanger sequencing were used to genotype a 676-bp fragment of the *env* gene (the immunodominant region of gp41). The details of the RT-PCR and sequencing procedures have been described previously (40). If the *env* RT-PCR failed, alternative primers specific for *env* were used. For rare subtypes, RT-PCR for *gag* p24 (468 bp) and *pol* integrase (864 bp) was also performed.

### Subgenomic sequence classification.

Subgenomic sequences were phylogenetically subtyped by analyzing genotypes and comparing them with the sequences in a comprehensive reference data set. This data set includes 480 whole-genome reference sequences that were used by Tongo and colleagues (12) to investigate the deep genetic diversity of HIV-1 group M. Five additional whole-genome sequences were added to the alignment, including three whole-genome subtype J and H sequences from Angola (8) and two additional unclassified whole-genome sequences (GenBank accession numbers KP718920 and KP718929). Briefly, this reference data set includes (i) representative whole-genome group M HIV-1 sequences of major subtypes (subtypes A to K) and subsubtypes (i.e., subsubtypes A1 and A2 and subsubtypes F1 and F2), (ii) representative whole-genome group M HIV-1 sequences of CRFs 01 to 72, (iii) all whole-genome HIV-1 sequences that the LANL database classifies as problematic sequences, (iv) and all whole-genome HIV-1 sequences that are listed as unclassified within the LANL database, including the two whole-genome sequences belonging to putative subtype L (last date of access, 1 May 2016).

Subgenomic sequences were aligned against homologous segments of the 485 reference sequences by use of the ClustalW program. Alignments were manually edited by use of the Geneious (v8.0.5) bioinformatics tool until a perfect codon alignment was achieved for each data set. A maximum likelihood (ML) tree topology for each of the alignments was inferred by use of the RAxML (v8.0.0) program (41) under the general time-reversible (GTR) model of nucleotide substitution (42) and the estimated gamma shape parameter (43). The fast parametric bootstrap resampling method (*n* = 1,000) was implemented on a 12-core Mac Pro computer to infer the support for splits. Each tree topology was visualized with the FigTree (v1.4.2) program (http://tree.bio.ed.ac.uk/software/figtree) and manually annotated.

Genotypes clustering within a subtype or CRF clade and with bootstrap values of >70 were classified as belonging to that particular clade. Sequences that were basal to a particular clade were analyzed for recombination by the use of Simplot software (44), and unique recombinants were classified as URFs. Sequences of *gag*, *pol*, and *env* with different classifications were also classified as URFs. Sequences that did not branch with any reference sequences or that clustered with unclassified reference sequences were considered unclassified.

To make an easily visualized tree for Fig. 2, a neighbor-joining phylogenetic tree and bootstrap values were inferred using the Phylip (v3.5c) software package (45).

### HIV-SMART NGS.

Eighteen isolates with remaining volume belonging to rare viral subtypes or isolates that exhibited signs of intersubtype recombination were targeted for whole-genome sequencing by the HIV-SMART NGS method. HIV-SMART libraries were prepared, sequenced, and analyzed as previously described (19). Briefly, the six-primer HIV-SMART mix was used, and reverse transcription reactions were performed at 42°C. HIV-SMART optimization experiments compared different reverse transcription temperatures of 47°C and 50°C, as well as the use of the Pico SMART cDNA kit (Clontech) and the addition of a nucleic acid concentrator step (Zymo Research) and a sizing column purification step (Clontech) to the standard HIV-SMART protocol. Isolate NGSID 10 (viral load, 5.01 log_10_ copies/ml) was diluted 1:100 in normal human plasma for the library preparation optimization experiments to create a sample containing 3 log_10_ copies/ml. All libraries were fragmented, barcoded, multiplexed, and sequenced on a MiSeq (Illumina) instrument as previously described (19). For samples with multiple NGS data sets from optimization experiments, all reads from conditions with an RT temperature of 42°C were used to generate a consensus genome sequence. NGS data were processed as previously described using CLC Genomics Workbench (v8.0) software (CLC Bio software; Qiagen) with minor modifications (19). Briefly, fastq data files were imported into CLC Genomics Workbench (v8.0) software and trimmed for quality and ambiguity, and the SMART primer adapter sequence was removed. For optimization experiments, reads were aligned only to the HIV-1 strain HXB2 reference genome for consistency in making comparisons. For the building of genomes, reads were aligned to 6 to 10 HIV subtype and CRF reference genomes, and complete genomes were built by aligning the resulting consensus sequences. The reference sequences used for read mapping are summarized in Table S1 in the supplemental material. For consensus sequences with gaps, contigs generated by *de novo* assembly were kept if they aligned to the consensus genome and were merged with NGS data in Sequencher (v5.2.3) software to create a final consensus sequence. Sanger sequencing was used to fill in the remaining small gaps (<200 bp) with the use of primer sequences specific for regions with full NGS coverage flanking the gap. The raw NGS data were realigned to the final genome sequence to generate NGS coverage and read mapping statistics. Open reading frames were confirmed and annotated with SeqBuilder (DNAStar Lasergene, v11.2) software.

### Genome sequence classification.

Complete whole-genome sequences were initially subtyped with two online subtyping tools: (i) the jumping profile hidden Markov model (jpHMM; http://jphmm.gobics.de) and (ii) the REGA (v3.0) subtyping tool (http://regatools.med.kuleuven.be/typing/v3/hiv/typingtool). Next, we subtyped the complete genomes through manual phylogenetic inference. The complete genomes were aligned against the 485 whole-genome reference and manually edited sequences as described above. An ML tree topology was inferred in the RAxML (v8.0.0) program (41) with the implementation of the GTR+G model of nucleotide substitution. The multiple or rapid bootstrap resampling method (*n* = 1,000) was implemented on a 12-core Mac Pro computer to infer the support for the splits. The final tree topology was visualized in FigTree (v1.4.2) software (http://tree.bio.ed.ac.uk/software/figtree) and manually annotated.

### Recombinant analysis.

Following the tree inference, complete genomes were scanned for recombination in the Recombination Detection Program, v4.0 (RDP4). Six different methods were used to scan for recombination using the default settings in RDP4. These six methods used RDP (46) and the GENECONV (47), Chimaera (48), MaxChi (49), Bootscan (29, 30), and SiScan (50) programs.

RDP recombination analyses were followed by manual scanning of whole genomes in Simplot (v3.5.1) software (44). Briefly, a subset of 120 full-genome HIV-1 sequences was selected from the 485 reference sequences. This subset broadly covered the global genetic diversity of HIV-1 group M subtypes, while it was enriched for genomes from the DRC and other sub-Saharan African countries. A list of the 120 HIV-1 strains is presented in Table S2. Additional reference sequences were added for the analyses of some NGSIDs on the basis of preliminary results of previous analyses. For example, for isolate NGSID 7, reference sequences belonging to CRF25 were included.

Recombinant fragments were extracted on the basis of the recombination breakpoints identified in the bootscan analyses. Recombinant fragments were subtyped through manual phylogenetic inference within the ML framework as described above. Trees of short recombinant fragments were visualized by the use of FigTree (v1.4.2) software (http://tree.bio.ed.ac.uk/software/figtree) and manually annotated.

The mosaic layout of each recombinant genome was annotated. The scheme for naming recombinants suggested by Tongo and colleagues (12) was used for the classification of recombinants. In this scheme, recombinant fragments clustering within established subtype or CRF clades of HIV-1 group M are designated with a capital letter (e.g., |A1| for subtype A1), while basal clustering to established subtypes or CRFs is designated by lowercase letters (e.g., |g| for subtype G). A recombinant that was not supported by bootstrap analysis (number of splits, <70) was designated unclassified (U).

### Accession number(s).

 The complete genome sequences of the various NGSID isolates have been deposited in GenBank under accession numbers KY392767 to KY392769 (NGSID 1 to NGSID 3, respectively), KY392770 to KY392773 (NGSID 5 to NGSID 8, respectively), KY392774 (NGSID 10), KY392775 to KY392779 (NGSID 12 to NGSID 16, respectively), and KY392780 (NGSID 18). The subgenomic sequences have been deposited in GenBank under accession numbers KY365010 to KY365181 (*env*), KY365182 to KY365202 (*gag*), and KY365203 to KY365216 (*pol*).

## Supplementary Material

Supplemental material
