# Supplementary material for: Sensitive Next-Generation Sequencing Method Reveals Deep Genetic Diversity of HIV-1 in the Democratic Republic of the Congo
Source: J Virol. 2017 Feb 28;91(6):e01841-16. doi: 10.1128/JVI.01841-16 (PMC5331799; doi:10.1128/JVI.01841-16)
Supplement: Supplemental material [file supp_91_6_e01841-16__index.html]

Sensitive Next-Generation Sequencing Method Reveals Deep Genetic Diversity of HIV-1 in the Democratic Republic of the Congo — Supplemental material 

# Sensitive Next-Generation Sequencing Method Reveals Deep Genetic Diversity of HIV-1 in the Democratic Republic of the Congo

## Supplemental material

- Supplemental file 1 -

  Fig. S1 (Genome coverage plots.)

  Fig. S2 (Recombinant profile of NGSID 6 as determined through manual phylogenetic inference of recombinant fragments.)

  Fig. S3 (Recombinant profile of NGSID 7 as determined through manual phylogenetic inference of recombinant fragments.)

  Fig. S4 (Recombinant profiles of NGSIDs 8 and 10 as determined through manual phylogenetic inference of recombinant fragments.)

  Fig. S5 (Recombinant profile of NGSID 12 as determined through manual phylogenetic inference of recombinant fragments.)

  Fig. S6 (Recombinant profile of NGSID 18 as determined through manual phylogenetic inference of recombinant fragments.)

  Table S1 (Reference strains for NGS read mapping.)

  Table S2 (The 120 full-genome HIV-1 group M sequences used as references for Simplot, Bootscan, RDP4, and short-fragment phylogenetic characterization analyses.)

  PDF, 974K
