# Supplementary material for: Sensitive Next-Generation Sequencing Method Reveals Deep Genetic Diversity of HIV-1 in the Democratic Republic of the Congo
Source: J Virol. 2017 Feb 28;91(6):e01841-16. doi: 10.1128/JVI.01841-16 (PMC5331799; doi:10.1128/JVI.01841-16)
Supplement: Supplemental material [file JVI.01841-16_zjv999182422s1.pdf]

Supplementary Figure 1 - Genome coverage plots. The number of reads (y-axis) per nucleotide position in each genome (x-axis) was determined for the final consensus genome for each specimen.

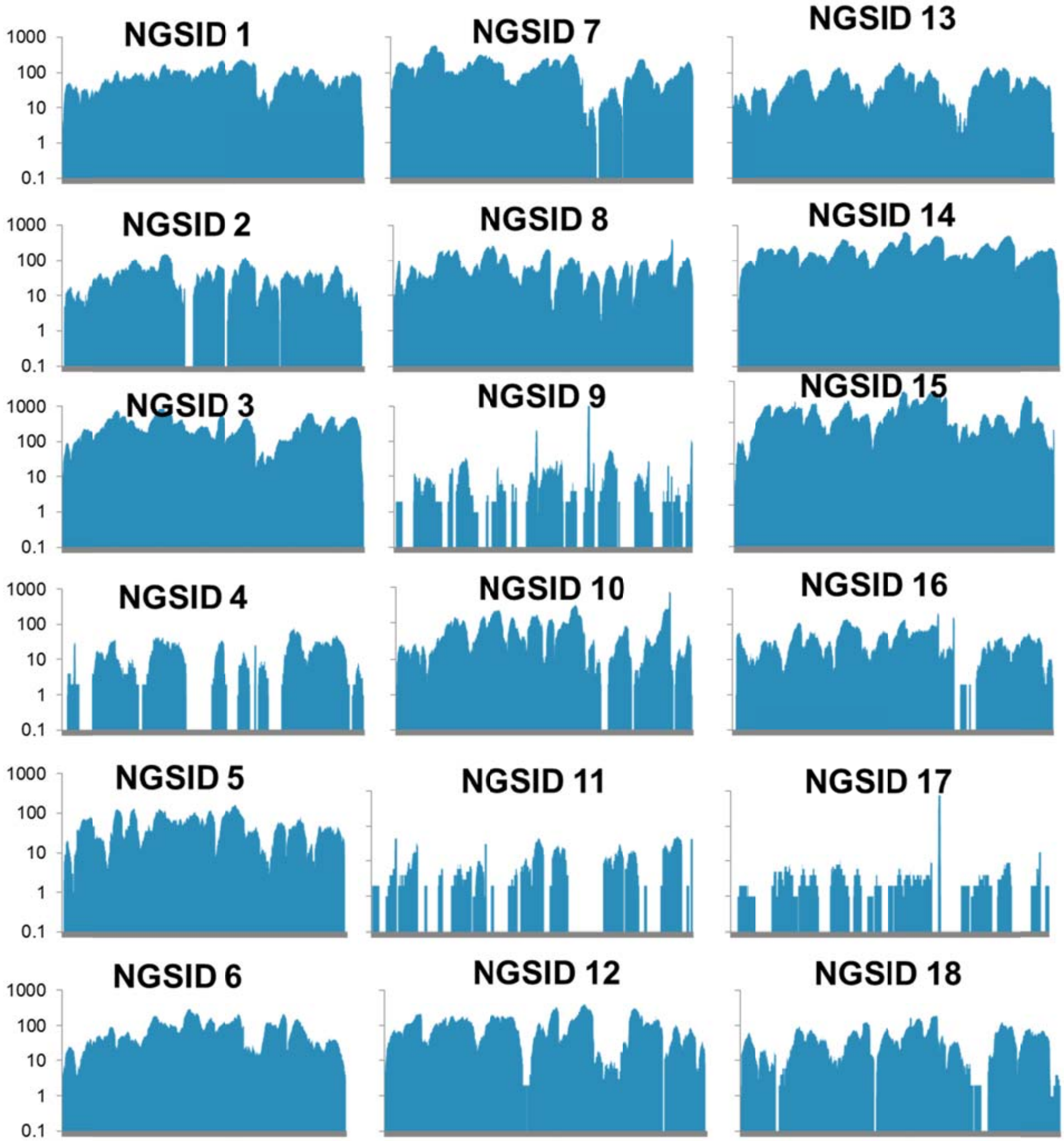

Supplementary Figure 2 - Recombinant profile of NGSID 6 as was determined through manual phylogenetic inference of recombinant fragments. Bootstrap support values for important branches are indicated. The recombinant mosaic layout of the genome of NGSID 6 was constructed with the Recombinant Drawing Tool from the LANL database. All coordinates are relative to the HXB2 reference strain. NGSID 6 was identified with the following recombination profile: A1-g-A1-g-h-H-G

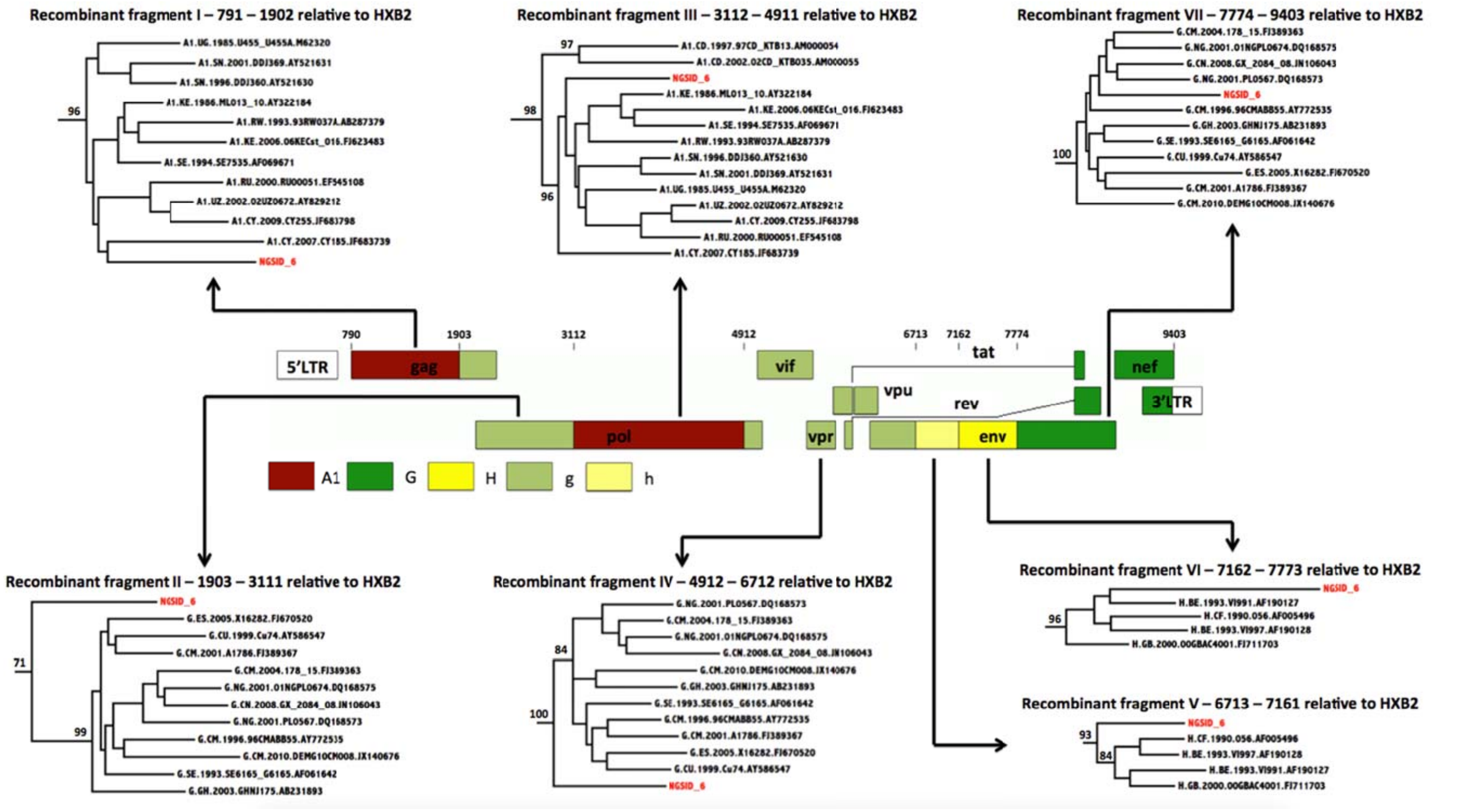

Supplementary Figure 3 - Recombinant profile of NGSID 7 as was determined through manual phylogenetic inference of recombinant fragments. Bootstrap support values for important branches are indicated. The recombinant mosaic layout of the genome of NGSID 7 was constructed with the Recombinant Drawing Tool from the LANL database. All coordinates are relative to the HXB2 reference strain. NGSID 7 was identified with a recombinant between CRF25 and subtype A1.

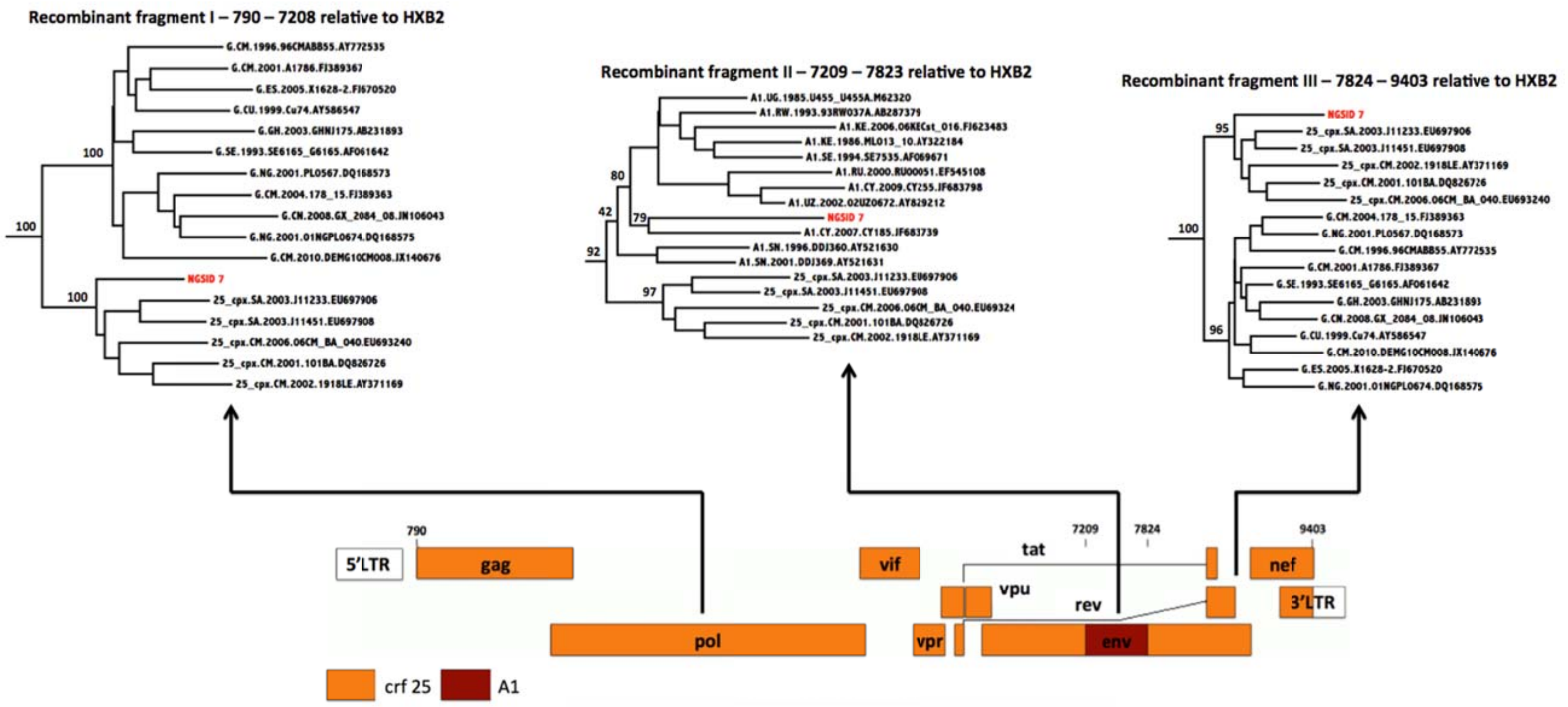

Supplementary Figure 4 - Recombinant profile of NGSIDs 8 and 10 as was determined through manual phylogenetic inference of recombinant fragments. Bootstrap support values for important branches are indicated. The recombinant mosaic layout of the genome of these two NGSIDs from the DRC was constructed with the Recombinant Drawing Tool from the LANL database. All coordinates are relative to the HXB2 reference strain. These two isolates were identified as a unique recombinant with the following recombination profile: A1-k-a1-h-g-h, with small lettering indicative of basal clustering to main clades.

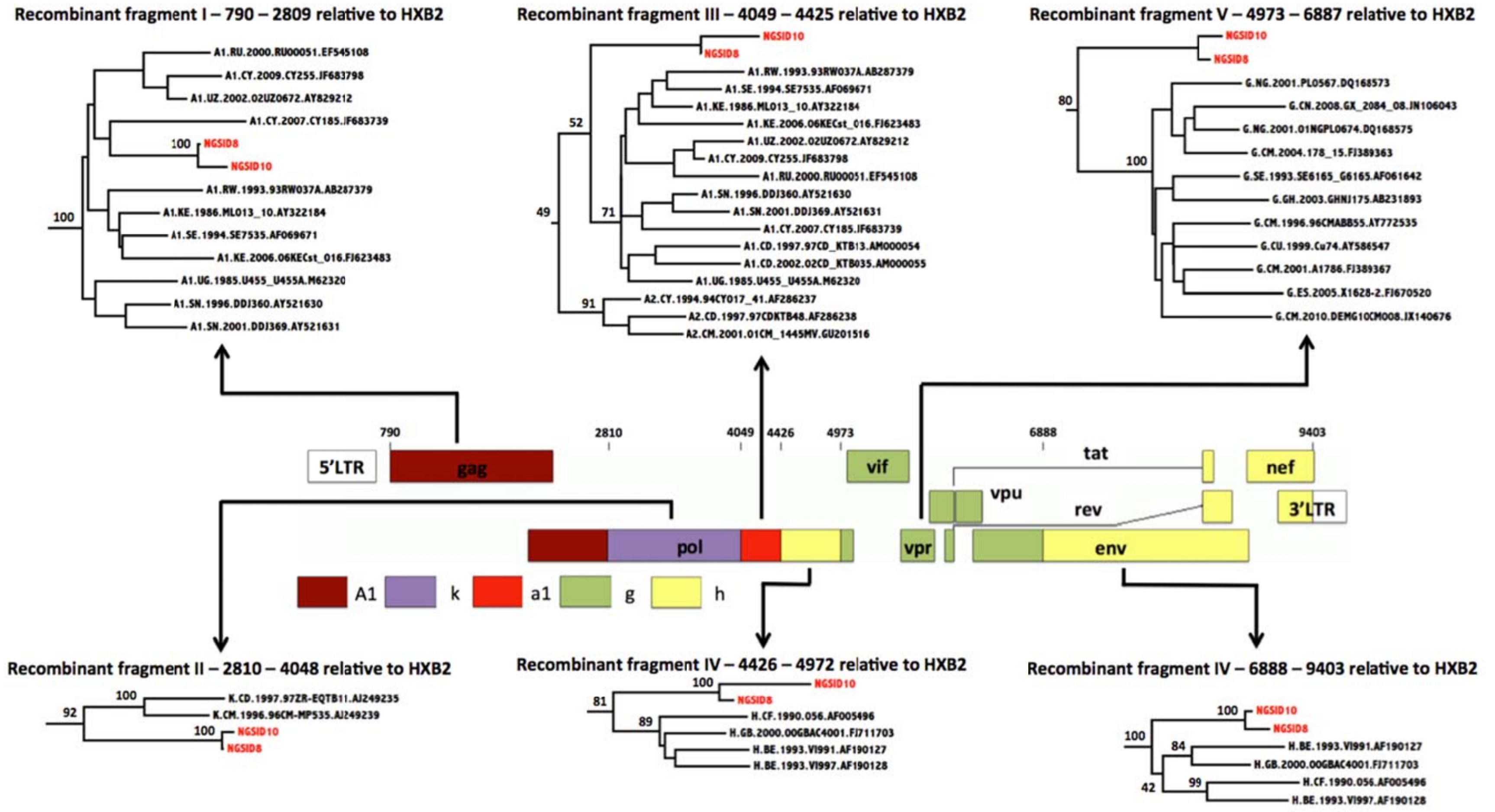

Supplementary Figure 5 - Recombinant profile of NGSID 12 as was determined through manual phylogenetic inference of recombinant fragments. Bootstrap support values for important branches are indicated. The recombinant mosaic layout of the genome of NGSID 12 was constructed with the Recombinant Drawing Tool from the LANL database. All coordinates are relative to the HXB2 reference strain. NGSID 12 was identified with a unique recombinant with the following profile: K-f-k-U-k-U, with small letters indicating basal clustering to main clades.

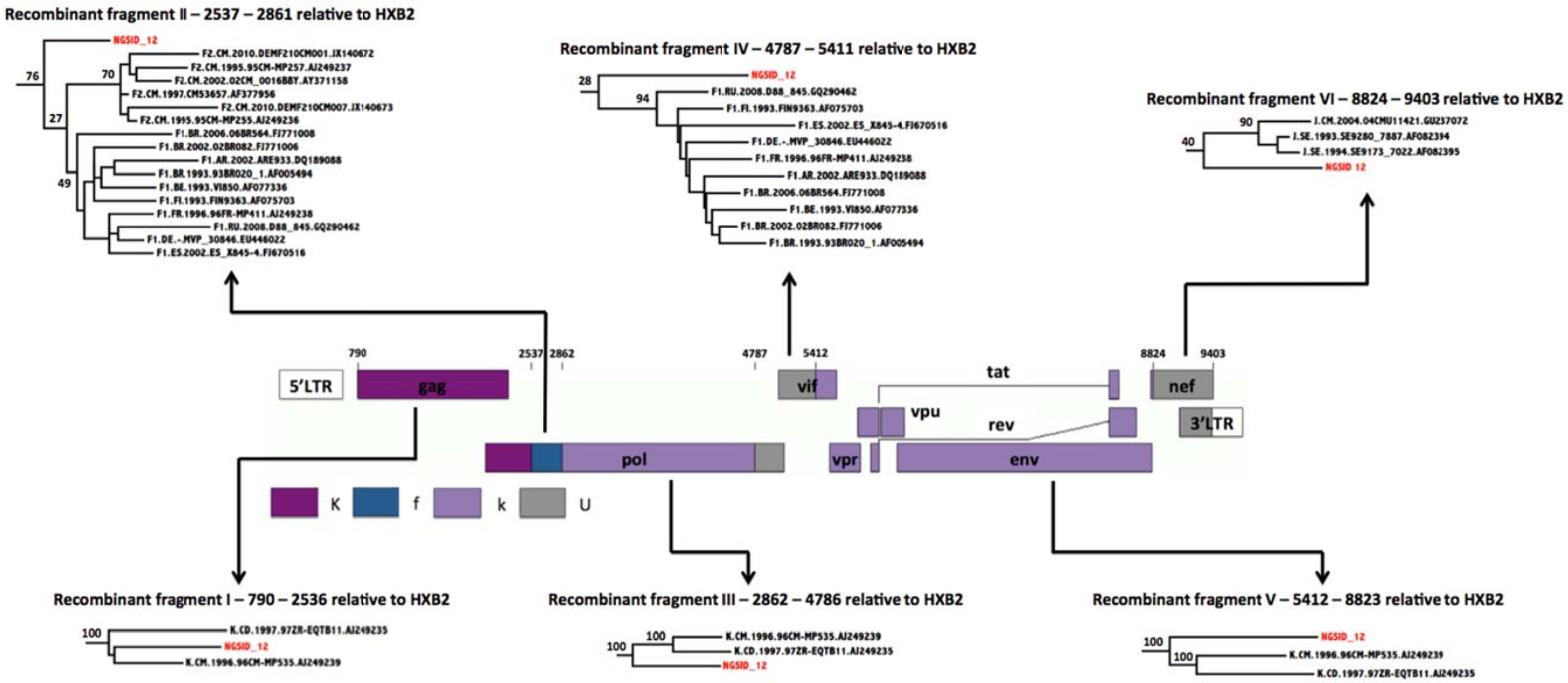

Supplementary Figure 6 - Recombinant profile of NGSID 18 as was determined through manual phylogenetic inference of recombinant fragments. Bootstrap support values for important branches are indicated. The recombinant mosaic layout of the genome of NGSID 18 was constructed with the Recombinant Drawing Tool from the LANL database. All coordinates are relative to the HXB2 reference strain. NGSID 18 was identified with a unique recombinant with the following profile: A1-k-A1-j-A, with small letters indicating basal clustering to main clades.

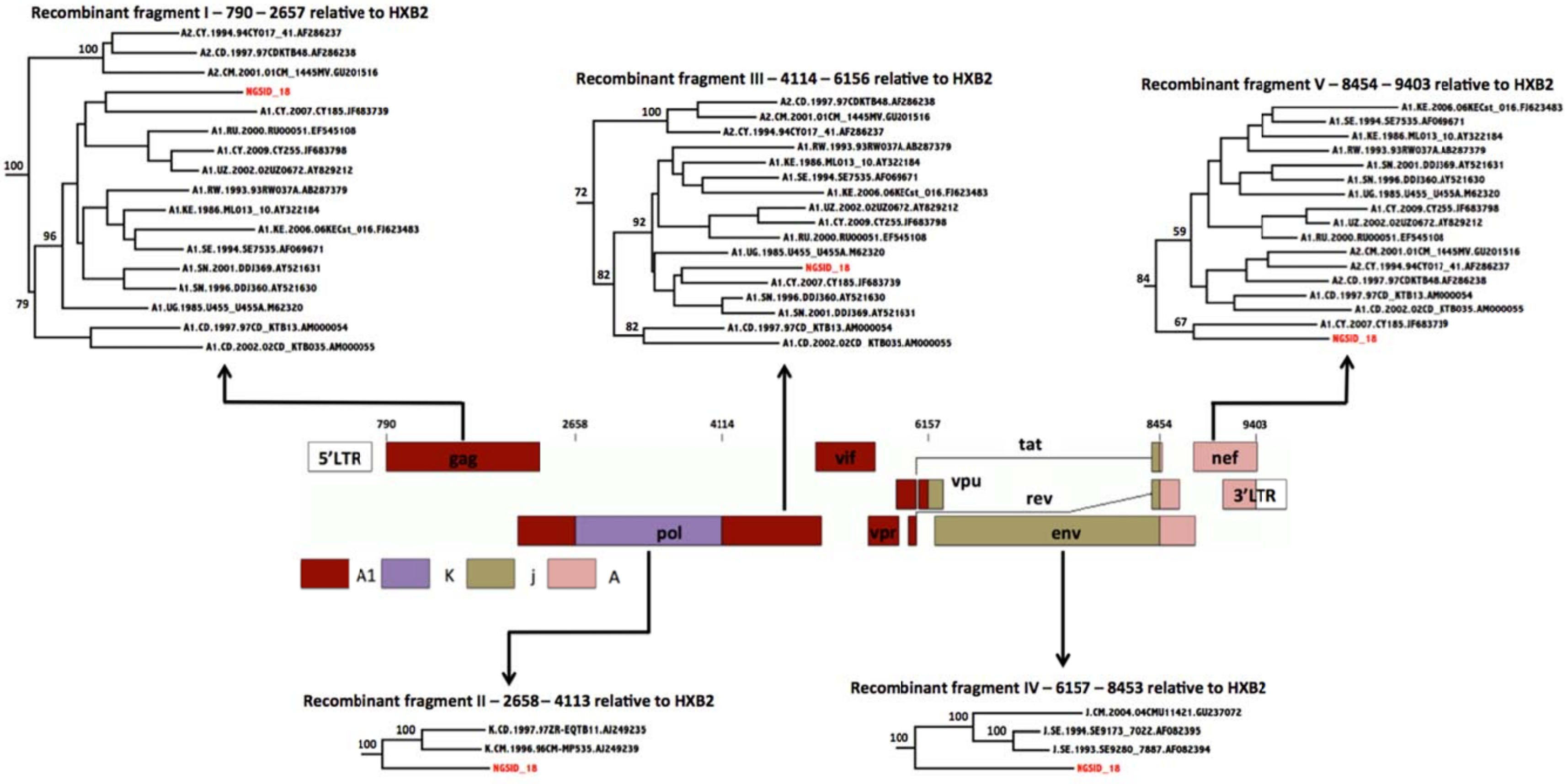

Supplementary Table 1 - Reference strains for NGS read mapping. The reference strains that were used to align the NGS reads for each genome consensus build are listed as <subtype>-<Genbank accession number>-<two letter country code>. Preliminary Simplot recombination analysis and Sanger sequencing in the *gag*, *pol*, and/or *env* regions were used to guide the selection of reference genomes for mapping the NGS reads. When multiple sequences were available for a subtype, selections were made to include the longest references and sequences from countries near the DRC.

NGSID 1	NGSID 2	NGSID 3	NGSID 4	NGSID 5	NGSID 6
C-AY228557-ZA	C-U46016-ET	D-K03454-CD	F1-AF075703-FI	F1-AB485659-RO	G-AF084936-CD
C-AB023804-IN	C-AY228557-ZA	D-DQ054367-KR	F1-FJ771006-BR	F1-FJ771006-BR	G-AY772535-CM
C-AB485643-DJ	C-KU168263	D-AY773338-ZA	F1-GQ290462-RU	F1-DQ979025-ES	G-AY612637-PT
C-AY901971-ZA	C-AB023804-IN	D-M22639	F1-DQ979025-ES	F1-AF075703-FI	06-AJ288981-SE
C-KU168262	C-KU168262	D-AJ320484-UG	F1-AB485659-RO	F-AF005494-BR	G-AY586549-CU
C-U46016-ET	C-AY901971-ZA	C-AY901971-ZA	F2-AF377956-CM	F2-AF377956-CM	G-AB485662-KE
C-AF286227-ZA	C-AB254142-ZM	B-GU733713-US	F-AF005494-BR	F2-AJ249236-CM	G-AB231893-GH
C-KU168263	C-AF286227-ZA	B-HXB2	B-HXB2	K-AJ249239-CM	B-HXB2
B-HXB2	C-AB485643-DJ	B-AY423387-NL		B-HXB2	02-KU168310-CM
	B-HXB2	B-U63632-US			

NGSID 7	NGSID 8	NGSID 9	NGSID 10	NGSID 11	NGSID 12
G-AB231893-GH	H-AF190127-BE	H-AF005496-CF	H-NGSID14-CD	K-AJ249235-CD	J-GU237072-CM
G-AB485662-KE	H-FJ711703-GB	H-AF190127-BE	F1-FJ771006-BR	K-AJ249239-CM	J-EF614151-CD
G-AY586549-CU	H-KU168279-CF	H-FJ711703-GB	C-AB023804-IN	J-AF082395-SE	J-AF082394-SE
G-AY612637-PT	H-AF005496-CF	H-KU168279-CF	H-AF005496-CF	L-AF286236-CD	K-AJ249239-CM
G-AY772535-CM	43-EU697904-SA	C-NGSID1-CD	H-KU168279-CF	C-NGSID1-CD	K-AJ249235-CD
G-AF084936-CD	02-KU168310-CM	A1-AF413987-UA	H-FJ711703-GB	L-AF457101-CD	L-AF457101-CD
02-KU168310-CM	B-HXB2	A2-AF286238-CG	H-AF190127-BE	B-HXB2	G-AY772535-CM
06-AJ288981-SE		A3-AY521630-SN	B-HXB2	J-GU237072-CM	B-HXB2
B-HXB2		A4-AM000053-CD	F2-AF377956-CM	J-AF082395-SE	
		A-U51190-UG			
		B-HXB2			

NGSID 13	NGSID 14	NGSID 15	NGSID 16	NGSID 17	NGSID 18
G-AY772535-CM	C-AY228557-ZA	C-AY228557-ZA	C-AY228557-ZA	A2-AF286238-CG	J-EF614151-CD
B-HXB2	J-AF082395-SE	J-AF082395-SE	J-AF082395-SE	A3-AY521630-SN	K-AJ249239-CM
J-AF082395-SE	C-AB023804-IN	C-AB023804-IN	C-AB023804-IN	A1-AF413987-UA	J-GU237072-CM
J-GU237072-CM	H-AF190127-BE	H-AF190127-BE	H-AF190127-BE	B-HXB2	B-HXB2
J-EF614151-CD	H-AF005496-CF	H-AF005496-CF	H-AF005496-CF	A-U51190-UG	J-AF082395-SE
J-AF082394-SE	H-FJ711703-GB	H-FJ711703-GB	H-FJ711703-GB	H-AF005496-CF	A2-AF286238-CG
K-AJ249239-CM	H-KU168279-CF	H-KU168279-CF	H-KU168279-CF	H-FJ711703-GB	A3-AY521630-SN
	B-HXB2	B-HXB2	B-HXB2	H-KU168279-CF	02-KU168310-CM
				A4-AM000053-CD	A3-AY521629-SN
					A1-AF413987-UA

Supplementary Table 2 - The 120 full-genome HIV-1 Group M sequences that were used as references for Simplot, Bootscan, RDP4 and short fragment phylogenetic characterization analyses. The analyses for NGSID 7 were supplemented with additional whole genome sequences of CRF25. Similarly the analyses for NGSIDs 8 and 10 were supplemented with additional whole genome sequences of CRF04, while analyses for NGSID 18 were supplemented with additional whole genome sequences of CRF45.

Subtype A	Subtype B	Subtype C	Subtype D	Subtype F	Subtype G	Other
A1.BY.AF193275	B.AR.AY037268	C.BR.AF286228	D.BR.KJ787683	F1.AO.FJ900266	G.CM.AY371121	H.BE.AF190127
A1.CD.AM000053	B.AU.AF042100	C.BR.U52953	D.CD.A07108	F1.AO.FJ900267	G.CM.AY772535	H.BE.AF190128
A1.CD.AM000054	B.CA.AY314044	C.BW.AF110959	D.CD.A34828	F1.AO.FJ900268	G.CM.FJ389363	H.CF.AF005496
A1.KE.AY322184	B.FR.A04321	C.CN.AY967806	D.CD.M22639	F1.AR.DQ189088	G.CM.FJ389365	H.GB.FJ711703
A1.SE.AF107771	B.GB.AJ271445	C.ET.U46016	D.CD.U88822	F1.BR.AB485656	G.CM.FJ389367	J.CM.GU237072
A1.SN.AY521629	B.HK.FJ460501	C.IN.AB023804	D.CM.AY371155	F1.BR.AY173957	G.CM.JX140676	J.SE.AF082394
A1.TZ.AF361872	B.HT.EU839600	C.KE.AY945738	D.CM.AY371156	F1.ES.FJ670516	G.CM.KP718915	J.SE.AF082395
A1.UA.AF413987	B.JP.AB731663	C.MW.AY713413	D.CM.AY371157	F1.FI.AF075703	G.CM.KP718923	K.CD.AJ249235
A1.UG.AB253428	B.KR.KJ140266	C.SN.AY713416	D.CM.JX140670	F1.FR.AJ249238	G.CM.KP718925	K.CM.AJ249239
A1.UZ.AY829212	B.NL.AY970946	C.SO.AY713415	D.KE.AF457090	F1.RO.AB485658	G.CU.AY586549	
A1.ZA.KJ948658	B.TH.AY173951	C.TZ.AF286234	D.KE.AY322189	F2.CM.AF377956	G.ES.AF423760	
A2.CD.AF286238	B.TT.EU839610	C.TZ.KC156220	D.SN.AB485648	F2.CM.AJ249236	G.ES.AF450098	
A2.CM.GU201516	B.TW.AF086817	C.US.AY444800	D.TD.AJ488926	F2.CM.AJ249237	G.ES.EU786670	
A2.CY.AF286237	B.US.AY835754	C.ZA.AF286227	D.TD.AJ488927	F2.CM.AY371158	G.ES.GU362882	
	B.US.AY835759	C.ZA.AY463217	D.TZ.AY253311	F2.CM.JX140672	G.GH.AB231893	
	B.US.AY835770	C.ZM.AB254150	D.UG.AB485650	F2.CM.JX140673	G.KE.AB485662	
	B.ZA.FJ647145	C.ZM.AB485645	D.UG.AY713418		G.KE.KF716477	
		C.ZM.AF286225	D.ZA.AY773338		G.NG.DQ168573	
		C.ZM.KP109496	D.ZA.AY773339		G.NG.DQ168575	
			D.ZA.AY773340		G.NG.DQ168576	
			D.ZA.AY773341		G.NG.U88826	
			D.ZA.EF633445		G.SE.AF061642	
					G.ZA.KJ948662	
